# Editorial: Interplay between oncomicrobes, the microbiota and the immune system: impact on responses to cancer immunotherapy

**DOI:** 10.3389/fimmu.2023.1269020

**Published:** 2023-08-15

**Authors:** Stephanie M. Dorta-Estremera, Esther A. Peterson, Josué Pérez-Santiago, JoAnn M. Sekiguchi

**Affiliations:** ^1^ Microbiology and Medical Zoology Department, University of Puerto Rico, San Juan, Puerto Rico; ^2^ Division of Cancer Biology, Comprehensive Cancer Center, University of Puerto Rico, San Juan, Puerto Rico; ^3^ Department of Biology, University of Puerto Rico, San Juan, Puerto Rico; ^4^ Department of Human Genetics, University of Michigan Medical School, Ann Arbor, MI, United States; ^5^ Department of Internal Medicine, University of Michigan, Ann Arbor, MI, United States

**Keywords:** microbiota, immune responses, oncomicrobes, cancer immunotherapy, microbiome, cancer

The tumor microenvironment is comprised of a complex milieu of stromal, immune, and tumor cells, in addition, commensal and opportunistic microorganisms that may exert local and systemic effects impact the tumor microenvironment and may modulate cancer initiation, progression, and cancer therapy efficacy. These communities of microorganisms (microbiota) may promote carcinogenesis and inflammation, thus, many studies are now elucidating the role of the microbiome on oncogenesis, immune responses, and treatment efficacy in cancer patients. In this Research Topic “*Interplay between Oncomicrobes, the Microbiota and the Immune System: Impact on Responses to Cancer Immunotherapy*”, different authors discuss how microorganisms are associated with oncogenesis, immune checkpoint inhibitor (ICI) efficacy, and ICI-mediated colitis, and also provide insights into animal model systems that may be more appropriate to study these relationships.

Only 12 microorganisms, including *Helicobacter pylori* (*H. pylori*) and human papillomavirus (HPV), are considered human carcinogens. As Oster et al. discuss, *H. pylori* infection has been associated with poor response to immunotherapies and anti-tumoral immune responses. *H. pylori* may induce the generation of regulatory T cells and type 2 macrophages, known to impair CD8+ cytotoxic T cell responses and thus, prevent tumor elimination.. Therefore, the identification of *H. pylori* in cancer patients may be a prognostic biomarker, and targeting *H. pylori* may improve the efficacy of ICI. Although *H. pylori* infection is associated with microbiota changes, these changes do not seem to play a major role in the efficacy of cancer immunotherapies. In contrast, HPV infection in the cervix has been associated with lower levels of *Lactobacillus* species along with a diverse microbiota, a marker of dysbiosis in the cervix. While the link between cervical microbiota, HPV infection, and cancer is being actively investigated, the role of anal microbiota in the development of HPV-related malignancies remains understudied. In this Research Topic, Elnaggar et al. identified differences in the anal microbiota related to HPV infection, dysplasia, and anal cancer. Although the study included a small cohort of patients, increased abundance of anorectal *Peptoniphilus*, *Fusobacteria*, *Porphyromonas*, and *Prevotella* were found in anal cancer subjects. Interestingly, similar bacteria have been associated with a pro-inflammatory microenvironment in gastrointestinal cancers. Additional studies are needed to better understand the connection between the anal microbiota, HPV colonization, and immune responses.

Most microbiome research has been focused on bacteria, and therefore less is known about the fungal communities (mycobiota) in cancer. The mycobiota is highly diverse and heterogeneous among urinary systems, and it may have an active role during the development and progression of bladder cancer (Li et al.). Li et al. performed a comprehensive analysis of the expression of C-type lectin receptors (CLRs), which have been recognized as innate pathogen-associated receptors for the mycobiota in bladder cancer. They found a significant association of CLRs with immune infiltration and immune-checkpoint receptors. Based on this study, the authors hypothesized that the urinary mycobiome plays a key role in the pathogenesis of bladder cancer and proposes more research on CLR-mediated anti-fungal immunity as a novel target for immunotherapy in bladder cancer and potentially other cancers.

Although ICIs are a promising treatment for various cancers through their stimulation of T cell-mediated anti-tumor immune responses, only a few patients obtain long-term clinical benefits and many develop immune-related adverse effects (irAEs). Interestingly, *Akkermansia muciniphila*, *Bifidobacterium pseudolongum*, and *Lactobacillus* in the gut have been associated with improved function of CD8+ T cells and better response to ICI (Zhou et al.). Moreover, *Bifidobacterium* and *Lactobacillus* have been associated with reduced ICI-mediated colitis (Han et al.). Conversely, *Bacteroides thetaiotaomicron*, *Escherichia coli*, and *Anaerotruncus colihominis* were associated with lower CD8+ T-cell tumor infiltration and no response to ICI. Thus, after the literature review performed by Han et al., they concluded that *Bifidobacterium* and *Lactobacillus* may have clinical relevance since these bacteria enhance anti-tumor efficacy of ICIs and reduce ICI-mediated colitis. However, more studies are needed to improve our understanding of the roles of these (and other) microbes on ICI responses and iRAEs development.

The restoration of the local microbiota and the utilization of microorganisms as therapeutics are important areas of research to improve the prognosis of cancer patients as discussed by Zhou et al.. Several studies have shown that fecal microbiota transplantation (FMT) is safe and feasible, and it can restore the gut microbiota by increasing the abundance of *Lactobacillus*. Moreover, FMT from ICI-responding patients into refractory metastatic melanoma patients improved clinical responses to ICI therapy and alleviated immune-mediated colitis (1, 2). Additional alternatives to restore the microbiota may be using probiotics, engineered bacteria, bacterial metabolites and/or dietary interventions. For example, the intake of fibers and polyphenol-rich berries may enhance ICI antitumor activity. There is a clear need for clinical trials that utilize microbiota modulators to test ICI efficacy and iRAEs.

One major hurdle in developing microbiota-targeted treatments is that most of the preclinical studies are performed in mouse models. Mice have a gut microbiota very dissimilar to humans and do not share similar environmental exposures, which may challenge the clinical research translation from mice to humans. For this reason, Kleber et al. discuss the use of canine models to decipher the potential of the microbiome as a diagnostic and therapeutic target in cancer. Importantly, canine ICIs are commercially available and FMT is an accepted practice in veterinary medicine; thus, canine models provide an opportunity to better understand gut microbial alterations and long-term effects after ICIs. Results from these types of studies may not only impact cancer treatment in humans but also dogs.

In conclusion, the microbiota holds significant potential to be used as a prognostic and therapeutic target. However, many important questions remain unanswered regarding how microbiota can be precisely shaped to improve the prognosis of cancer patients and increase the efficacy of ICIs while decreasing iRAEs. To answer these key questions, it is essential to examine the impact of the multitude of factors that regulate the microbiome, including environmental factors and diet in a controlled setting such as using large animal models.

## Author contributions

SD-E: Writing – original draft, Writing – review & editing. EP: Writing – review & editing. JP-S: Writing – review & editing. JS: Writing – review & editing
